# Did Wheat Breeding Simultaneously Alter Grain Concentrations of Macro- and Micro-Nutrient Over the Past 80 Years of Cultivar Releasing in China?

**DOI:** 10.3389/fpls.2022.872781

**Published:** 2022-03-30

**Authors:** Baozhen Hao, Jingli Ma, Luyao Si, Lina Jiang, Xiaojie Wang, Chong Yao, Siyuan Ma, Chunxi Li, Zhiqiang Gao, Zhimin Wang

**Affiliations:** ^1^School of Life Sciences and Basic Medicine, Xinxiang University, Xinxiang, China; ^2^College of Life Sciences, Henan Normal University, Xinxiang, China; ^3^Ministerial and Provincial Co-innovation Centre for Endemic Crops Production with High-Quality and Efficiency in Loess Plateau, Shanxi Agricultural University, Taigu, China; ^4^College of Agronomy, China Agricultural University, Beijing, China

**Keywords:** Chinese wheat landrace, cultivar, grain micronutrient concentration, macronutrient, grain yield, dilution effect, genetic variation, year of release

## Abstract

Biofortification of wheat with mineral through crop breeding is a sustainable and cost-effective approach to address human mineral malnutrition. A better understanding of the trends of grain concentrations of mineral nutrients in wheat over the breeding period may help to assess the breeding progress to date. A 2-year field experiment using 138 Chinese wheat landraces and 154 cultivars was conducted. Grain concentrations of micronutrients (Cu and Mn) and macronutrients (N, P, and K) were measured and corrected for a yield level to elucidate the trends of these mineral nutrients over the 80 years of cultivar releasing and identify genetic variation for these mineral nutrients in cultivars and landraces. Large genetic variation exists for grain mineral nutrients concentrations among tested genotypes, indicating that selection for enhancing mineral nutrient concentrations in wheat is possible. Landraces showed a slightly wide genetic variation of grain Cu concentration and a much narrow variation of Mn concentration when compared to modern cultivars. Grain concentrations of Cu and Mn decreased slightly with increasing grain yield with a weak correlation, while N, P, and K concentrations declined obviously with increasing yield with a strong correlation, revealing that increased grain yield had a strong negative effect on grain concentration of macronutrients, but a relative weak negative effect on micronutrients concentrations. When considering the impact of the variation in yield on mineral concentrations, grain concentrations of Cu, Mn, N, P, and K in wheat cultivars released from 1933 to 2017 exhibited different trends with a year of variety release. Grain Cu, N, and P concentrations showed significant decreasing trends over a breeding period, while grain Mn and K concentrations showed no clear trend, suggesting wheat breeding in China over the past 80 years has decreased grain concentrations of Cu, N, and P, and did not alter Mn and K concentrations. Finally, a total of 14 outstanding accessions with high grain mineral nutrients concentrations/contents were identified, and these genotypes can be considered as promising donors for developing mineral-dense wheat cultivars.

## Introduction

Mineral malnutrition presents a serious global challenge to humankind and affects approximately 40% of the world’s population, mostly pregnant women, infants, and pre-school children (Amiri et al., 2018). Globally, the most widely mineral deficiencies are zinc (Zn) and iron (Fe), and there are 1.1 and 2.3 billion people in the world at risk of Zn and Fe deficiency, respectively (Wessells and Brown, 2012; Kassebaum et al., 2014; Kumssa et al., 2015). Dietary copper (Cu) and manganese (Mn) deficiency is increasing among human population across the developed and developing countries (Welch and Graham, 2002; Velu et al., 2017). A survey in China indicated that 20% of primary school children in Guangdong region are at risk of inadequate Cu intake (Yang et al., 2007). In addition, a previous study showed that dietary intake of Cu in the UK has fallen significantly from 1986 to 2000 (Fan et al., 2008). In Finland, it is reported that, *per capita*, daily intakes of Cu decreased by 20% and Mn by 14% during the 1970s to the 2000s (Ekholm et al., 2007). Biofortification has shown great potential for addressing micronutrient malnutrition in human populations (White and Broadley, 2009). It has been widely recognized that biofortification strategy should focus on the staple food crops, such as wheat (*Triticum aestivum* L.), which is a staple food for over 35% of the world’s population, and provides 20% of daily dietary protein, 21% of calories, and 14% of Cu, 11% of Zn, 13% of magnesium (Mg), and 15% of Fe (Fan et al., 2008; Li et al., 2022). Wheat was found to contain relatively lower concentration of essential micronutrients (e.g., Fe, Zn, Cu, and Mn) in the grain (Balint et al., 2001; Liu et al., 2021). Therefore, increasing grain mineral nutrient concentration in wheat is considered as a promising strategy to diminish human micronutrient malnutrition around the world.

Conventional and modern plant breeding is advocated as a sustainable and cost-effective approach to increase mineral nutrient concentration in wheat grains (Graham et al., 1999; Bouis and Saltzman, 2017). The success of wheat breeding largely relies on the amplegenetic variation for the target trait, which requires large germplasm resources (White and Broadley, 2005; Amiri et al., 2015). Wheat landrace was found to have substantial diversity and higher concentration of Zn and Fe in the grain relative to cultivated wheat, and has been identified as potential germplasm resources for breeding new genotypes with high mineral nutrient concentrations (Velu et al., 2014, 2018; Khokhar et al., 2020). However, little is known about genetic diversity among wheat landraces for Cu and Mn concentrations in the grain. China has more than 4,600-year history of wheat cultivation, and there are large sets of natural germplasm resources, such as landraces (Hao et al., 2008), which have been used to generate new wheat varieties with improved resistance or tolerance for biotic and abiotic stresses (Hao et al., 2008; Lin et al., 2019). The grain Zn and Fe concentrations and their genetic variations in Chinese wheat landraces have been evaluated in previous work (Hao et al., 2021). However, little information is available about grain Cu and Mn concentrations in Chinese wheat landraces, and evaluating Cu and Mn levels of those landraces may help to identify useful genetic resources for developing micronutrient-dense cultivars.

Grain yield of major cereal crops increased steadily during the twentieth century due to crop breeding and improved agronomic management (Grassini et al., 2013), which was accompanied by significant decrease in grain concentration of mineral nutrient (Calderini et al., 1995). For example, several previous studies evaluated grain Cu and Mn concentrations in wheat varieties released during different eras and found that the concentrations of Cu and Mn have decreased over the years (Ekholm et al., 2007; Fan et al., 2008; Murphy et al., 2008). The similar declined trends were also observed for grain concentrations of Zn and Fe in different types of wheat germplasm (Fan et al., 2008; Zhao et al., 2009; Morgounov et al., 2013; Amiri et al., 2015; Guttieri et al., 2015). In addition, it is reported that grain macronutrient concentrations (e.g., N and P) have been declined over the breeding period (Calderini et al., 1995; Calderini and Ortiz-Monasterio, 2003). However, results from other studies showed that grain concentration of mineral nutrient in wheat may not always be negatively correlated with grain yield or kernel weight, and produced either no clear trend or even a trend toward slight increase throughout the history of breeding (Graham et al., 1999; Chen et al., 2017; Liu et al., 2021). The inconsistency among these results may be related to grain a yield level. The comparison of mineral nutrients among genotypes in these studies was performed using raw concentration data without considering the impact of the variation in grain yield on mineral concentration. It has been proposed that, to interpret and exploit the genetic variation for mineral nutrient in the grain, any possible concentration and dilution effects caused by grain yield capacity of genotypes should be considered (McDonald et al., 2008). Therefore, elucidating the trends of grain mineral nutrient concentration in wheat over the breeding period without taking into account the yield capacity of genotypes will not necessarily identify the actual effect of wheat breeding on mineral nutrient concentration. In previous work using the same set germplasms, grain Zn and Fe concentrations were analyzed by regressing Zn or Fe concentration against grain yield and then evaluating the residuals of such relationships against a year of variety release, and Zn and Fe in the grain were found to show different trends over a year of release (Hao et al., 2021). This finding indicates that the effect of wheat breeding on mineral nutrient concentration might depend on the element as documented by Calderini and Ortiz-Monasterio (2003). Cu and Mn are essential micronutrients for human health, while little is known about the effect of Chinese wheat breeding on grain Cu and Mn concentrations over the 80 years of cultivar release. Also, it is not known how the Chinese wheat breeding over the past years affects macronutrient (N, P, and K) concentrations when considering the impact of yield on mineral nutrient concentration. The objectives of the current study were to (1) to compare the differences in micronutrients (Cu and Mn) and macronutrients (N, P, and K) concentrations between Chinese wheat landraces and modern cultivars; and (2) to elucidate changes and trends in micronutrients (Cu and Mn) and macronutrients (N, P, and K) concentrations over decades due to breeding.

## Materials and Methods

### Plant Material

A set of 292 wheat accessions (*Triticum aestivum* L.; hexaploid genome = AABBDD), including 154 cultivars released from 1933 to 2017 and 138 Chinese landraces (Supplementary Table 1), were studied in this 2-year field experiments. All of these landraces were from the Chinese wheat mini-core collection (Hao et al., 2008). Out of 154 cultivars, 95 were the Chinese wheat mini-core collection, and the remaining 59 released after 1990 were the most popular varieties of their time in the main wheat–growing areas of China. In addition, based on the time of release, these154 cultivars were categorized into two groups: pre-Green Revolution cultivars that were released during 1933–1970 (*n* = 55) and post-Green Revolution cultivars that were released during 1971–2017 (*n* = 99).

### Site Description and Field Experiments

A 2-year field trial was carried out at Xinxiang Experimental Station (Xinxiang, Henan Province, China, 35.2°N, 113.8°E) in 2017–2018 (2018) and 2018–2019 (2019) seasons. The soil texture at the experimental location is a clay loam. The soil alkaline hydrolysis N, available K, Olsen P, and organic matter contents in a 0–30-cm layer are 65.1 mg kg^–1^, 112.7 mg kg^–1^, 9.1 mg kg^–1^, and 12.2 g kg^–1^, respectively. As determined, using the protocol of Lindsay and Norvell (1978), the diethylenetriamine pentaacetic acid (DTPA)-extractable Cu and Mn concentrations were 1.92 and 9.73 mg kg^–1^ of soil, respectively. Seasonal precipitation for the 2018 and 2019 wheat growing season was 215 and 192 mm, respectively, and climate data during the study period were obtained from the agro-meteorological station next to the study field.

A randomized complete block design with three replications was used in each season. Each experimental plot consisted of 2 m^2^ (1.0-m wide and 2.0-m long with 0.20 m between rows). Wheat seeds were sown using a hand-operated seeder that can adjust seeding rates to achieve a target plant density of 300 plants m^–2^ for each variety. Starter fertilizers were applied at planting at rates of 100 kg N ha^–1^, 108 kg P_2_O_5_ ha^–1^, and 109 kg K_2_O ha^–1^, and 50 kg N ha^–1^ was applied at a stem elongation stage followed by irrigation. The plots were watered using movable pipelines at the stages of seeding (100 mm), jointing (75 mm), and anthesis (75 mm). The control of insects and weeds was implemented by applications of herbicide and insecticides, respectively.

### Grain Sampling and Chemical Analysis

Three center rows in each plot were harvested manually to determine grain yield and yield components, including kernels per spike and kernel weight. Grain yield measured in 2018 and 2019 seasons and yield components measured only in the 2018 season were adjusted to 13% moisture. A subsample of about 100 g of grain was obtained and rinsed with tap and deionized water, respectively, and then dried in an oven at 65°C until constant weight, ground, and storage for further chemical analysis.

To determine Cu, Mn, and potassium (K) in a grain sample, 0.2 g of each ground sample was soaked with 5-ml digestion mixture (4.0-ml HNO_3_ + 1.0-ml H_2_O_2_) and then digested using microwave-accelerated reaction system (CEM, Mars 6, Matthews, NC, United States). Copper, Mn, and K concentrations in the digested solutions were determined by Inductively Coupled Plasma Optical Emission Spectrometry (ICP-OES, iCAP 7000, Thermo Fisher Scientific, Germany). For quality control, reagent blanks and certified standard reference material (No. GBW10011) were included in the analyses. For Cu, Mn, and K, the averaged recovery rates were more than 95%, and relative standard deviation (RSD) was less than 5%. For the analysis of grain nitrogen (N) and phosphorus (P), each ground sample was digested with H_2_SO_4_-H_2_O_2_, and N and P concentrations in the digested solutions were measured using a high-resolution digital colorimeter auto-analyzer (AA3, SEAL Company, Germany; United States). The measurements of grain N, P, and K concentrations were canceled in the second season due to COVID-19. The concentrations of Cu, Mn, N, P, and K in the grain were expressed in terms of oven-dried weight.

### Statistical Analysis

Descriptive statistical analysis was performed using the R function “hist” in R software (R Core Team, 2019). Histograms were calculated for grain Cu, Mn, N, P, K concentrations. ANOVA was conducted using the PROC MIXED procedure in SAS (SAS Institute Inc, 2009) to evaluate each factor. More details of ANOVA are given in our previous paper (Hao et al., 2021). To evaluate the trends of grain concentrations and yield of mineral nutrients (Cu, Mn, N, P, and K) over a year of release, for Cu and Mn, trait deviation calculated from the mean of all genotypes within an experiment was used (Maeoka et al., 2020), and for N, P, and K (measured in one season), trait values were used. Six models (linear, quadratic, cubic, piecewise, logistic, and sigmoidal) were used to determine deviation data (or trait value) as a function of a release year. Models were fitted with GraphPad Prism 8 (GraphPad Software Inc., La Jolla, United States), and the final model was determined by Akaike information criterion (AIC) and coefficient of determination (*R*^2^). Grain yield is a critically important determinant of grain nutrient concentration (Guttieri et al., 2015); thus, the relationships between the deviations (or trait values) of grain element concentrations and grain yield in cultivars (*n* = 154) and all wheat accessions (*n* = 292) were assessed using linear or non-linear regression, and then evaluated the residuals of such relationships against a year of release/landrace vs. cultivar (Lollato et al., 2019; Maeoka et al., 2020). Pearson correlation analysis was calculated using the R function “chart.Correlation” to assess the direct linear relationships among the traits that were measured in the 2018 season. In addition, to further visualize the associations among measured traits, principal component analysis (PCA) was performed based on correlation matrix, and the R function “princomp” was used for the PCA. The traits used in PCA analysis were grain concentrations of Cu, Mn, N, P, and K, and grain yield, kernel weight, and kernels per spike.

## Results

### Variation in Nutrient Concentrations

**FIGURE 1:**
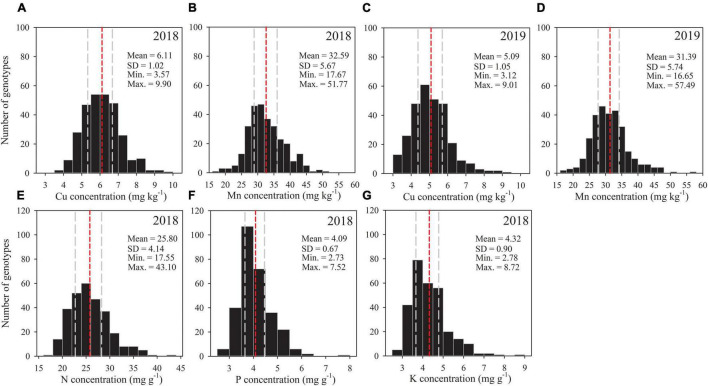
Frequency distribution of 292 wheat accessions for grain concentrations of **(A,C)** Cu, **(B,D)** Mn, **(E)** N, **(F)** P and **(G)** K in the 2018 and 2019 field experiments. Statistical distribution parameters are shown in upper right. Gray dashed lines indicate 25 and 75% quantiles, and the red dashed line indicates the mean for each trait.

A large genotypic variation was found for all grain mineral nutrients among tested genotypes in each season (Figure 1). The genotypic variation of Cu was about 2.8-fold in each season, and the variation of Mn was 2.9-fold in the first season and 3.5-fold in the second season (Table 1). In the case of N, P, and K, genotypic variation was 2.5-, 2.8-, and 3.1-fold, respectively. As average of all genotypes, grain Cu concentration was significantly greater in the 2018 season (6.11 mg kg^–1^) than the 2019 season (5.09 mg kg^–1^), while Mn concentration was similar in both seasons (Figure 1).

**TABLE 1:** Grain concentrations of Cu, Mn, N, P, and K for wheat cultivars (*n* = 154) and landraces (*n* = 138) in 2018 and 2019 growing seasons.

Traits	Seasons	Cultivars		Landraces	
		Mean	Range	Mean	Range
Grain Cu concentration (mg kg^–1^)	2018	6.01	4.30–9.39	6.22	3.57–9.90
	2019	4.97	3.12–9.01	5.23	3.22–8.60
Grain Mn concentration (mg kg^–1^)	2018	32.79	17.67–51.77	32.37	19.00–44.78
	2019	31.42	16.65–57.49	31.36	19.75–46.54
Grain N concentration (mg g^–1^)	2018	24.06	17.55–37.94	27.74	20.24–43.10
Grain P concentration (mg g^–1^)	2018	3.87	2.73–5.98	4.33	3.07–7.52
Grain K concentration (mg g^–1^)	2018	4.16	2.83–8.72	4.50	2.78–7.87

Averaged across seasons, grain Cu concentration for cultivars and landraces varied by 2.2- and 2.5-fold, ranging from 3.83 to 8.47 mg kg^–1^ (mean: 5.49 mg kg^–1^) and from 3.63 to 9.25 mg kg^–1^(mean: 5.73 mg kg^–1^), respectively (Table 1). In the case of Mn, grain concentration across seasons for cultivars varied by 3.1-fold, ranging from 17.16 to 53.65 mg kg^–1^, which was larger than that for landraces with 2.2-fold, ranging from 20.79 to 45.40 mg kg^–1^. The variation in N concentration for cultivars and landraces was about 2.1-fold. In the case of P, genotypic variation was higher for landraces (2.4-fold) than cultivars (2.2-fold). Grain K concentration for cultivars and landraces fluctuated by 3.1 and 2.8 times, respectively. These data indicate that the genotypic variation of Mn and K was greater for cultivars than landraces, but the variations of Cu and P were slightly wide for landraces, and the variation of N was similar between landraces and cultivars.

Maximum grain Cu concentration among all accessions was obtained from four cultivars (Dixiuzao, Zhemai1, Yanan11, and Taizhong23) and two landraces (Hongdongmai and Fumai) by more than 8 mg kg^–1^ (Supplementary Table 2). The highest grain Mn concentration was obtained from four cultivars (Taizhong23, Dixiuzao, Fengdecun5, and Zhengmai366) and one landrace (Zhahong) by more than 45 mg kg^–1^ (Supplementary Table 2). This indicates that the genotypes Dixiuzao and Taizhong23 were the superior in terms of both Cu and Mn contents in the grain, so they could be regarded as genotypes with strong genetic capacity in absorption and accumulation of Cu and Mn. Among cultivars, the genotype Kashibaipi had the highest N, P, and K concentrations with 37.94, 5.98, and 8.72 mg g^–1^, respectively. Among landraces, the genotypes Shanmai and Fumai had relatively high N concentration by more than 37 mg g^–1^; Shanmai, Fumai, Bailanghuimai, and Liuyuehuang showed high P concentration by more than 5.9 mg g^–1^; and Shanmai, Biantouguangkemai, and Banjiemang exhibited high K concentration by more than 7.0 mg g^–1^ (Supplementary Table 2).

### Trends of Grain Nutrient Concentrations and Yields

**FIGURE 2:**
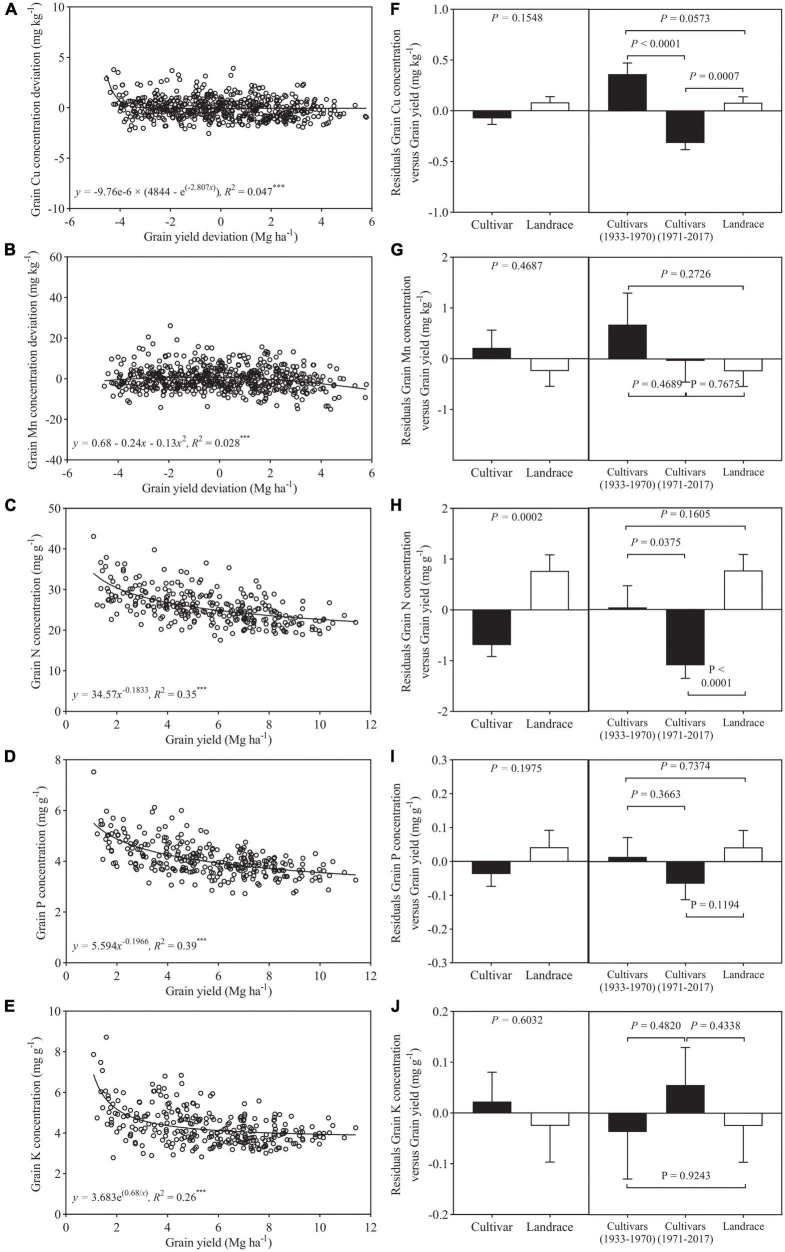
Relationship between grain yield deviation and grain concentration deviations of **(A)** Cu, **(B)** Mn, **(C)** N, **(D)** P, and **(E)** K for wheat cultivars and landraces (*n* = 292). Residuals of the regression as affected by wheat class **(F–J)** (bars show mean and standard error). Values correspond to the data of two seasons (2017–2018 and 2018–2019). *** is statistically significant at a *p* < 0.001 level.

Among 292 genotypes, a significant non-linear relationship was found between the deviations of grain Cu and Mn concentration and grain yield and also between N, P, and K concentration and grain yield (Figure 2). Grain Cu deviation decreased steeply at grain yield deviation less than −2.4 Mg ha^–1^ and then remained almost constant. Unlike Cu deviation, grain Mn deviation showed a slight increase at grain yield deviation less than −0.9 Mg ha^–1^ and then declined slowly (Figures 2A,B). Grain concentrations of N, P, and K always decreased with increasing grain yield (Figures 2C–E). The squares of the correlation coefficients (*r*^2^) between micronutrient concentration and grain yield were less than 0.05, which is much lower than that between macronutrient concentration and grain yield (0.26–0.39) (Figure 2), suggesting micronutrient concentration is far less sensitive to the increase of grain yield compared to macronutrient concentration.

In the case of Cu, Mn, P, and K, the residuals of the regression analysis of mineral nutrient concentration against grain yield did not differ significantly between cultivars and landraces, and, for N, the residual was significantly greater in landraces than cultivars (Figures 2F–J). In addition, the residuals from the relationships of Mn, P, and K concentrations and yield were not significantly different between landraces, pre- and post-Green Revolution cultivars. In the case of Cu and N, the residual was significantly lower in post-Green Revolution cultivars as compared to landraces and pre-Green Revolution cultivars (Figures 2F,H).

**FIGURE 3:**
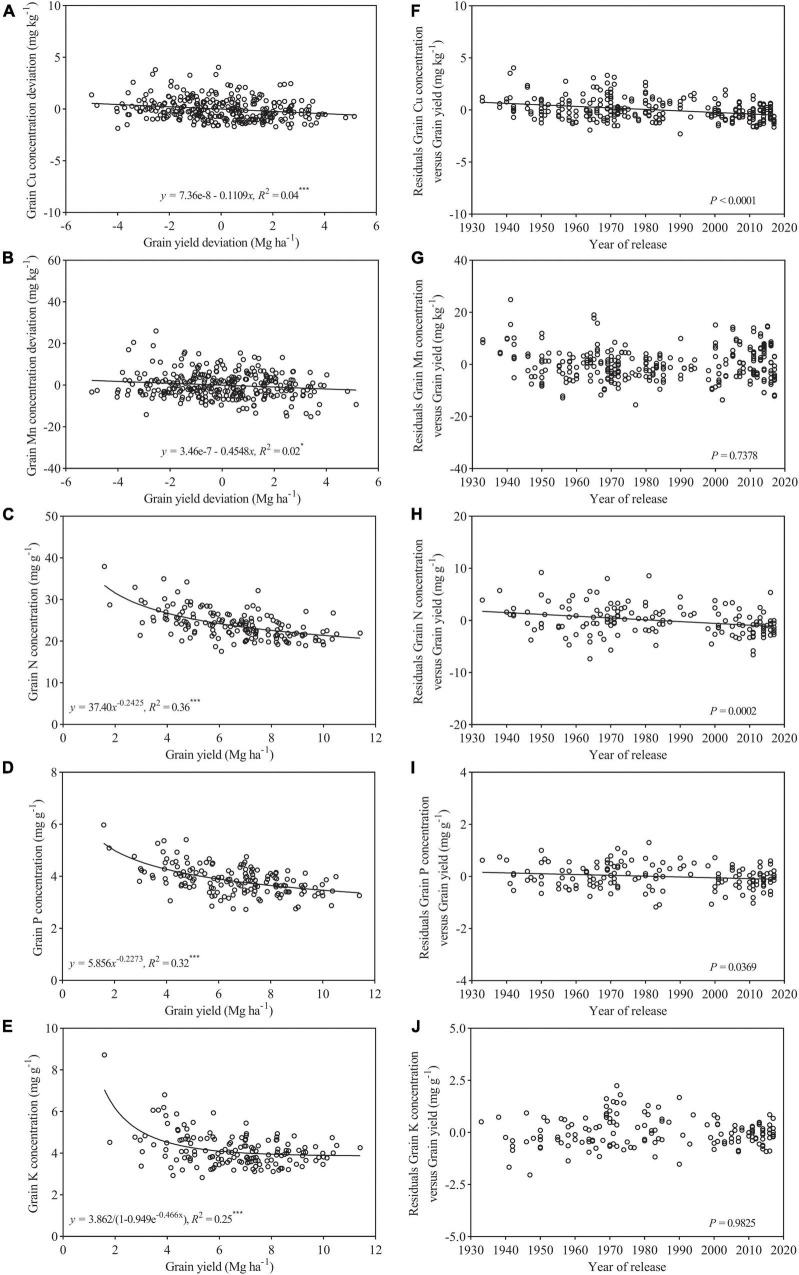
Relationship between grain yield deviation and grain concentration deviations of **(A)** Cu, **(B)** Mn, **(C)** N, **(D)** P, and **(E)** K for wheat cultivars released from 1933 to 2017 (*n* = 154). Residuals of the regression as affected by a year of genotype release **(F–J)**. Values correspond to the data of two seasons (2017–2018 and 2018–2019). * and *** are statistically significant at *p* < 0.05 and < 0.001 levels, respectively.

Within the subset of 154 cultivars released during 1933–2017, both grain Cu and Mn deviations showed slightly declined trends against grain yield deviations with a low *r*^2^ (0.02–0.04) (Figures 3A,B). There were obvious decreasing trends in grain concentrations of N, P, and K with increasing grain yield with high *r*^2^ (0.25–0.36) (Figures 3C–E). This suggests that increased grain had a strong negative effect on grain concentration of macronutrients, but a relative weak negative effect on micronutrients. Furthermore, in the case of Cu, N, and P, their residuals from the regression analysis showed a significantly declined trend with a year of release, but, for Mn and K, the residuals showed no clear trend over time (Figures 3F–J).

**FIGURE 4:**
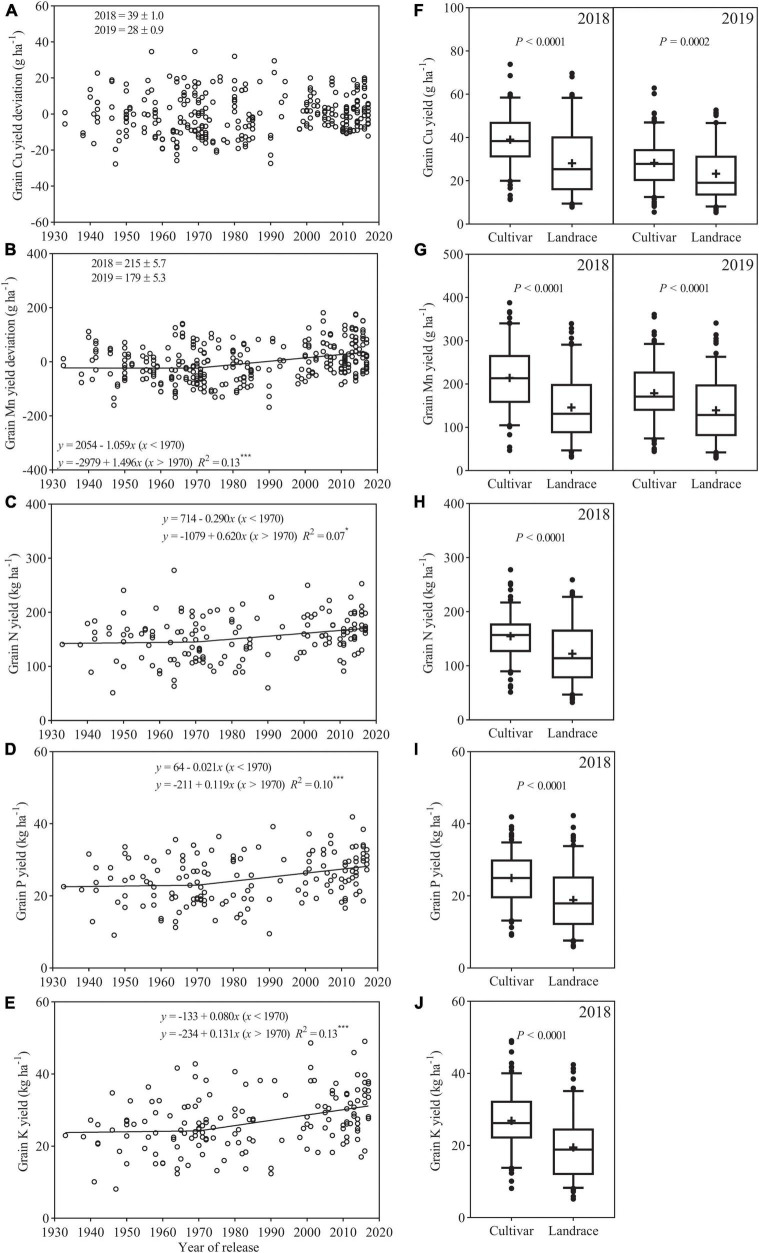
Relationships between the year of release and the grain yield deviation (or yield) of **(A)** Cu **(B)** Mn, **(C)** N, **(D)** P, and **(E)** K. Comparison of grain yield of **(F)** Cu, **(G)** Mn, **(H)** N, **(I)** P, and **(J)** K in the 2018 and 2019 field experiments. The boxes indicate the middle two quartiles; whiskers indicate the 95% confidence limits; circles indicate outliers; the solid horizontal line and the plus sign indicate median and mean, respectively. ns, not significant (*p* > 0.05), * and *** are statistically significant at *p* < 0.05 and < 0.001 levels, respectively.

Among the 154 cultivars, yield deviation of Cu showed no clear trend with a year of release (Figure 4A). Yield deviation of Mn showed bilinear relationship with a year of release and declined (−1.059 g ha^–1^ year^–1^) slightly between 1933 and 1970 and then increased at a higher rate (1.496 g ha^–1^ year^–1^) after 1970 (Figure 4B). Yields of N, P, and K showed bilinear relationship with a year of release and first remained an almost constant level of 148, 23, and 23 kg ha^–1^ and then increased at a rate of 0.620, 0.119, and 0.131 kg ha^–1^ year^–1^, respectively (Figures 4C–E). Cultivars showed greater Cu and Mn yields than landraces in each season (Figures 4F,G). Average N, P, and K yields for cultivars were 154.7, 25.0, and 26.9 kg ha^–1^, which were 26.5, 32.3, and 38.1% greater than that for landraces, respectively (Figures 4H–J).

### Relationships Between Traits

**FIGURE 5:**
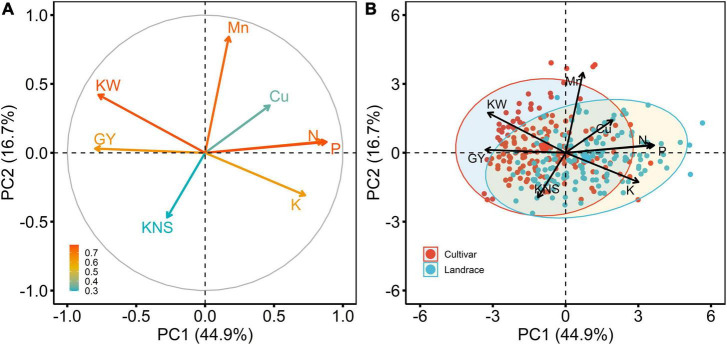
Principal component analysis (PCA) of eight variables estimated for 292 wheat accessions, including 154 cultivars and 138 landraces in the 2018 field experiment. **(A)**, projection of the model variables on the first two principal components (PC1 and PC2), and the arrows represent variables, which are colored by their quality of representation on the factor map, and a scale adjacent to the plot indicates the values of corresponding variables; **(B)**, genotype by variable biplots of 154 cultivars and 138 landraces with regard to eight variables. The variables include grain Cu concentration (Cu), grain Mn concentration (Mn), grain N concentration (N), grain P concentration (P), grain K concentration (K), grain yield (GY), kernels per spike (KNS), and kernel weight (KW).

PCA was performed to elucidate the relationships among the traits investigated in the 2018 season (Figure 5). The first two principal components (PC1 and PC2) explained 61.6% of the total variance. PC1 accounted for 44.9% of the data variation and clearly separated N, P, K, and Cu concentrations in its positive direction, while grain and kernel weight were separated in the negative direction. PC2 accounted 16.7% of the data variation, and this variation was caused mainly by Mn concentration and kernels per spike. Thus, with respect to PC1, the points located in the first and fourth quadrants present the highest of N, P, K, and Cu concentrations, and, with respect to PC2, the points presented in the second quadrant are those with the highest of Mn concentration (Figure 5A). Moreover, PCA indicated there were three trait groups in the plot; the first group was composed of grain N, P, and K concentrations, the second group consisted of grain Cu and Mn concentrations, and the third group contained grain yield, kernel weight, and kernels per spike (data not shown).

**FIGURE 6:**
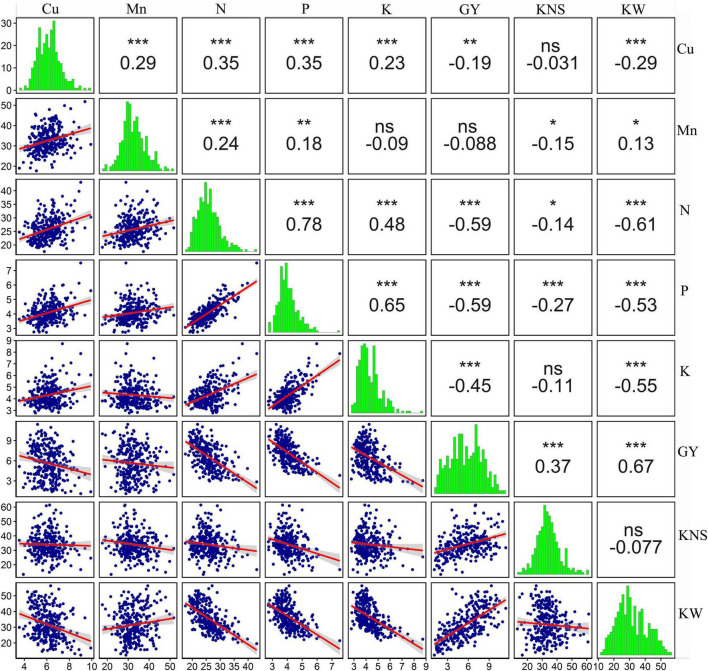
Pairwise comparison of all the variables measured in the 2018 field experiment. The upper-right panel shows estimated pairwise Pearson correlation coefficients. The diagonal panel shows histograms. The lower-left panel shows a scatter plot with a LM smoother added to aid visual interpretation. ns, not significant (*p* > 0.05), *, **, and *** are statistically significant at *p* < 0.05, *p* < 0.01, and *p* < 0.001 levels, respectively. The variables include grain Cu concentration (Cu), grain Mn concentration (Mn), grain N concentration (N), grain P concentration (P), grain K concentration (K), grain yield (GY), kernels per spike (KNS), and kernel weight (KW).

The associations among the traits could also be detected in PCA, for example, three macronutrients (N, P, and K) were strongly correlated with one another (Figure 5A), which was consistent with the findings of correlation analysis (*r* = 0.48–0.78, *p* < 0.001) (Figure 6). In addition, a significant positive association between Cu and Mn concentrations was detected (*r* = 0.29, *p* < 0.001) (Figure 6). Copper concentration was poorly correlated with kernels per spike (*r* = −0.031, *p* > *0.05*) and negatively correlated with grain yield and kernel weight (*r* = −0.19 and −0.29, respectively, *p* < 0.001). Manganese concentration was poorly correlated with grain yield, negatively correlated with kernels per spike, and positively correlated with kernel weight (Figure 6). Those results indicate that a yield level might be the main factor determining grain Cu concentration, and might only has a minor influence on grain Mn concentration. Furthermore, macronutrients (N, P, and K) showed strong and negative correlations (*r* = −0.45 to −0.59, *p* < 0.001) with grain yield, while a poor association (*r* = −0.088 and −0.19) between micronutrients (Cu and Mn) and yield was observed (Figure 6), indicating grain yield might showed different effects on mac- and micronutrients.

The differences in grain yield, yield components, and grain concentration of N, P, K, Cu, and Mn between landraces and cultivars were analyzed (Figure 5B). The biplot chart indicated that cultivars were mostly concentrated in the quadrants at the left side of the biplot, and in the direction of grain yield and kernel weight and the reverse direction of grain K, P, N, and Cu concentrations. The results indicated that the cultivars might have high values of grain yield and kernel weight and low grain K, P, N, and Cu concentrations. While most landraces were found in the quadrants at the right side of the biplot, suggesting landraces could obtain high grain K, P, N, and Cu concentrations and low kernel weight and grain yield.

## Discussion

### Variation in Mineral Nutrient Concentrations

Copper and Mn are essential micronutrients for human health and are generally lacking in human diets, and their grain concentrations in staple food crops, such as wheat, have been extensively reported. In this study, with 292 wheat accessions of which 138 were Chinese wheat landraces and 154 were modern cultivars, average Cu and Mn concentration in the grain was 5.60 and 31.99 mg kg^–1^, respectively. In Italy, Ficco et al. (2009) reported that average grain Cu and Mn concentrations in 84 durum wheat cultivars released since 1,900 were 7.41 and 47.96 mg kg^–1^, respectively. In a field experiment in China with 265 wheat genotypes, Zhang et al. (2010) found that Cu and Mn concentrations in the grain were 7.39 and 48.8 mg kg^–1^, respectively. In Siberia, Morgounov et al. (2013) revealed that grain concentration of Cu and Mn in 32 historical wheat varieties released during 1891–1994 was 2.74 and 38.73 mg kg^–1^, respectively. In addition, in the field trials conducted in the US with 299 wheat genotypes, including 193 cultivars and 106 breeding lines, average grain concentration of Cu and Mn across three trials was 3.74 and 42.17 mg kg^–1^, respectively (Guttieri et al., 2015). Pandey et al. (2016) reported that grain Cu and Mn concentrations in 150 wheat genotypes collected from Indians and Turkish were 5.9 and 36.0 mg kg^–1^, respectively. These findings indicate that Cu concentration in this study was in accordance with that reported in the abovementioned studies, whereas Mn concentration was lower than that in the abovementioned studies. In addition, Mn concentration in this experiment was in line with results of Batten (1994), who found that grain Mn concentration in wheat grown in Canada, Australia, the United States, and the United Kingdom was 42.9, 40.76, 39.13, and 23.2 mg kg^–1^, respectively.

Genotypic variation has been recognized as a key determinant of a genetic biofortification program (Gomez-Coronado et al., 2016). The results of our experiments showed that there was substantial genetic variation for concentration of Cu and Mn in wheat grains among 292 wheat genotypes. Wide genetic variation among wheat genotypes for the concentration of micronutrients (Cu and Mn) in the present study are in accordance with some former reported studies with 4.4-fold (1.42–6.20 mg kg^–1^) and 3.3-fold (23.0–76.6 mg kg^–1^) (Guttieri et al., 2015), 2.3-fold (5.3–12.1 mg kg^–1^) and 2.2-fold (32.2–70.6 mg kg^–1^) (Zhang et al., 2010), and 3.5-fold (4.5–15.6 mg kg^–1^) and 3.6-fold (18.1–65.6 mg kg^–1^) (Pandey et al., 2016), respectively. The different ranges of Cu and Mn concentrations between our and their results may be due to different genotypes and different experimental conditions.

Wheat landrace is generally considered as a potential germplasm resource and can be integrated into wheat breeding programs for enhancing the concentrations of essential mineral nutrients in the grain (Guzmán et al., 2014; Velu et al., 2014). Our results suggested that, compared to wheat cultivars, landraces had a slightly wider range of Cu concentration. Our findings were consistent with those of Ficco et al. (2009), who documented that grain Cu and Mn concentrations varied by 1.8-fold (5.9–10.4 mg kg^–1^) and 1.3-fold (43.0–57.8 mg kg^–1^) for wheat landraces and 2.4-fold (5.8–14.0 mg kg^–1^) and 1.4-fold (41.3–57.4 mg kg^–1^) for cultivars, respectively (Ficco et al., 2009). These findings together with the results obtained from our experiments indicate that Chinese wheat landraces do not show impressive genetic variation for grain concentrations of Cu and Mn as compared to cultivated wheat.

In addition, this study showed that the range and the average grain concentration of N were significantly different between landraces and cultivars. The variation in concentration of N for landraces was higher than that for cultivars, and average N concentration was 15% greater in landraces than cultivars. Among landraces, the genotypes Shanmai and Fumai have the highest N concentration of 43.10 and 39.79 mg g^–1^, which were higher than maximum N concentration among cultivars (37.94 mg g^–1^). Similarly, Amiri et al. (2018) also reported that wheat landraces exhibited higher grain protein concentration. These findings suggest that Chinese wheat landraces have high potential for use in breeding for increasing grain protein concentration of modern wheat cultivars. It has been reported that grain protein in cereal grains representing a sink for mineral elements and overexpressing metal-storage proteins by genetic engineering could improve the mineral content in grains (Rawat et al., 2013). High levels of available protein and amino acid seem more beneficial for enhancing bioavailability of mineral elements in the human body (Cakmak et al., 2010).

### Yield Dilution Effect on Mineral Nutrient Concentrations

**FIGURE 7:**
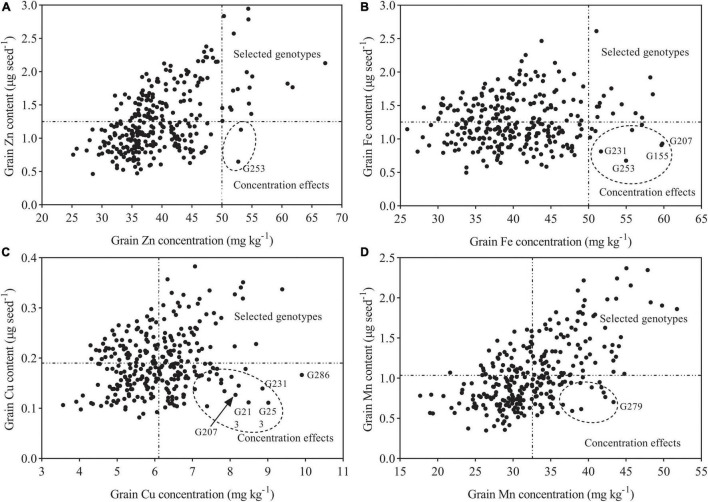
Graphical representation of concentration effects for Zn, Fe, Mn, and Cu. **(A)** Grain Zn content vs. Grain Zn concentration. **(B)** Grain Fe content vs. Grain Fe concentration. **(C)** Grain Cu content vs. Grain Cu concentration. **(D)** Grain Mn content vs. Grain Mn concentration. Dashed vertical lines represent a target level of genetic biofortification in wheat in **(A,B)**, and average mineral concentrations of the tested genotypes in **(C,D)**.

Relative low grain concentrations of mineral and protein in modern wheat cultivars relative to the older genotypes, primitive, and wild wheat have been reported (Amiri et al., 2015), which could be a consequence of a yield dilution effect (Grassini et al., 2013; Cakmak and Kutman, 2018). However, it is reported that wild emmer accessions contain extremely high Zn, Fe, and protein in the grain, and also show high grain yield, suggesting high nutrition concentrations in the grain were genetically controlled and not affected by yield dilution effect (Cakmak et al., 2000, 2010). Similarly, previous studies revealed that grain concentration of a mineral element in wheat may not always negatively correlate with grain yield or kernel weight (Graham et al., 1999; Liu et al., 2021), and Chen et al. (2017) reported that yield dilution effects on concentration of Zn in wheat grains may not necessarily happen. In addition, it is reported that the negative associations between yield and mineral concentration might be overcome by selection, and biofortification of staple crops with mineral elements can be reached without compromise on grain yield (White and Broadley, 2009). These findings indicate that grain nutrition concentration was mostly determined by not only yield dilution but also breeding strategy. In the present study, as shown in Figures 3, 7, both macronutrients (N, P, and K) and micronutrients (Cu and Mn) concentrations in the grain of 145 wheat cultivars declined with the increase in grain yield. A rapid decline with relatively higher *r*^2^ (0.25–0.36) was observed for N, P and K, and Cu and Mn exhibited a slight decrease with a very weak association (*r*^2^:0.02–0.04). These results indicated that the effect of yield dilution on mineral nutrients concentrations would depend on an element, and showed a strong negative effect on macronutrients but a relative weak negative effect on micronutrients. Our findings were consistent with those reported by Calderini and Ortiz-Monasterio (2003). Our results also revealed that the correlation was significantly negative between grain Cu concentration and kernel weight and grain yield, while grain Mn concentration showed weak association with these two traits (Figure 6). In a former study, grain yield and kernel weight were strongly and negatively associated with grain Fe concentration, and were poorly associated with grain Zn concentration (Hao et al., 2021). These results indicate that yield dilution effect is significant for Cu and Fe concentrations in wheat grains but is not significant for Mn and Zn concentrations. Similarly, it has been reported that dilution effect might be strong for one mineral element but relatively weak for another element (Ficco et al., 2009; Zhao et al., 2009). In the case of N, P, and K, their association with grain yield and kernel weight were similar, suggesting the effect of yield dilution on element concentration did not differ among N, P, and K.

### Trends of Grain Nutrient Concentrations Through the Breeding Period

The decreased trends in grain Cu and Mn concentrations in wheat with the breeding period have been previously documented (Ekholm et al., 2007; Fan et al., 2008; Murphy et al., 2008). The similar declined trends were also observed for grain concentrations of Zn and Fe in different types of wheat germplasm (Fan et al., 2008; Zhao et al., 2009; Morgounov et al., 2013; Amiri et al., 2015; Guttieri et al., 2015). In addition, a number of studies have shown that plant breeding has decreased grain N and P concentrations in modern wheat genotypes, which was related to continuous yield improvement throughout the history of breeding (Calderini et al., 1995; Ortiz-Monasterio et al., 1997). However, the comparison of mineral nutrient concentrations among genotypes in these studies was performed using raw concentration data without considering the impact of the yield variation on mineral nutrients concentration. It has been reported that yield dilution effect might show a large or even overriding effect on grain concentration of mineral nutrients, and to compare mineral nutrient concentration between genotypes without considering the grain yield of genotypes may lead to wrong conclusions (McDonald et al., 2008). In current the study, the changes and trends in grain nutrient concentrations over decades due to breeding were evaluated, and the analysis of the raw concentration data was corrected for a yield level, which could overcome the yield dilution effect and detect the true level of genetic variation in grain concentration of mineral nutrients. The results showed that grain concentration of Cu, Mn, N, P, and K in wheat cultivars released from 1933 to 2017 showed different trends with a year of variety release. Copper, N, and P concentrations decreased through the breeding period, while grain Mn and K concentrations showed no clear trend, suggesting Chinese wheat breeding over the past 80 years did not alter grain concentrations of Mn and K, but it decreased Cu, N, and K concentration. Similarly, in previous work using the same set germplasm and the same analytical method, grain Zn and Fe concentrations showed different trends with a year of release from 1933 to 2017 (Hao et al., 2021). Thus, the data from the current and previous studies reveal that, when considering the yield of genotypes, Chinese wheat breeding over the past 80 years showed different impacts on four types of micronutrients (Zn, Fe, Mn, and Cu), and has increased grain Zn concentration, decreased Fe and Cu concentration, and did not alter grain Mn concentration. In terms of macronutrients (N, P, and K), Chinese wheat breeding has decreased grain N and P concentrations, and did not alter K concentration in the grain. Furthermore, the results from this study and previous study (Hao et al., 2021) also showed that post-Green Revolution cultivars tend to have higher Zn but lower Cu, N, and Fe concentrations and similar Mn, P, and K concentrations as compared to landraces and pre-Green Revolution cultivars.

### Identification of Outstanding Genotypes

Taken together with our previous data, a substantial genetic variation for grain concentrations of Zn, Fe, Cu, and Mn in tested wheat genotypes was identified, which provides an opportunity to choose outstanding wheat genotypes regarding grain mineral concentration. Here, outstanding genotypes were identified according to the concentration (amount per unit weight) and content (total amount per one grain) of mineral nutrients (Gomez-Becerra et al., 2010). The outstanding genotypes for Zn and Fe were selected from those having mineral concentration higher than 50 mg kg^–1^ as suggested by Graham et al. (2007) and content that was more-than-average value of 292 genotypes (1.25 μg seed^–1^). The identification of superior genotypes for Cu and Mn was selected from those having concentration over 6.11 and 32.59 mg kg^−1^ (mean value for 292 genotypes) and content that was more than 0.19 and 1.04 μg seed^–1^ (mean value for 292 genotypes), respectively. Among 292 wheat genotypes, several genotypes (Pingyang27, Yangfumai101, and Jimai23) were among the best for Zn concentration and showed high-mineral content (2.57–2.79 μg seed^–1^) (Supplementary Table 2 and Figure 7). A high Zn concentration of 56.16 mg kg^–1^was also observed for the genotype Shanmai, while its Zn content was much lower (0.65 μg seed^–1^), and this might be associated with its smaller grain weight (12.32 mg seed^–1^), which may lead to “concentration effects.” High-grain concentration of mineral nutrients in wheat caused by “concentration effects” as a result of small grain size or weight has been reported in a previous study (Gomez-Becerra et al., 2010). Furthermore, concentration effects may have also been occurred on the genotype Huoliaomai, which had the maximum concentration of Zn (63.25 mg kg^–1^) and Fe (66.98 mg kg^–1^) among the tested genotypes across seasons but relatively low contents of Zn (1.37 μg seed^–1^) and Fe (1.22 μg seed^–1^) (Supplementary Table 2 and Figure 7). In the case of Fe, the genotypes Banjiemang, Fumai, and Hongpixiaomai were in the top ranking in both seasons, while their high grain Fe concentrations were the result of “concentration effects” due to the low grain weight (15–16 mg seed^–1^) (Supplementary Table 2 and Figure 7). The genotypes Changzhi6406, Yanan11, Fengkang2, and Beijing8 with high mean Fe concentrations across seasons seemed to have unstable performance. The other genotypes among the top 20 accessions showed high Fe concentration (over 50 mg kg^–1^), but their Fe contents were relatively low due to medium grain weight (20–30 mg seed^–1^). These results indicate that it is difficult to choose a superior genotype with both high concentration/content and good stability. Therefore, more experiments should be carried out in the future to evaluate grain mineral nutrients (especially Fe) of these 292 wheat genotypes under different environments.

The grain Cu concentrations of the top 3 genotypes (Hongdongmai, Dixiuzao, and Zhemai1) in Supplementary Table 2 were near or higher than 8.5 mg kg^–1^, of which the genotype Diuxiuzao seemed to be less stable, and the other two showed relatively low Cu contents (approximately 0.2 μg seed^–1^). Moreover, high-grain Cu concentrations of the genotypes Banjiemang, Fumai, and Hongqiangchang in both seasons were the results of “concentration effects” caused by small grain weight (13–16 mg seed^–1^). The genotypes Liying5, Enmai4, and Yanan11 were among the best for Cu concentration in both seasons and showed higher Cu concentrations and contents. The genotypes (Dixiuzao, Taizhong23, Zhoumai27, Fengdecun5, Zhengmai366, Shannong14, and Zhengmai101) were among the best for Mn concentration in each season and had high mineral content (1.86–2.37 μg seed^–1^) and high grain weight (35.94–52.62 mg seed^–1^) (Supplementary Table 2 and Figure 7). In the case of Zn, Fe, Cu, and Mn, the genotype Taizhong23 was common in the top ranking across years and had relatively high grain mineral contents, but it seemed to be less stable regarding Zn, Fe, and Cu concentrations. The genotype Niuzhijia had high-average grain concentrations of Zn, Fe, and Mn across seasons and was stable for Fe and Mn, while it did not have high grain weight (29.02 mg seed^–1^). Finally, according to the concentration and content of mineral nutrients, a total of 14 outstanding wheat genotypes (Pingyang27, Yangfumai101, Jimai23, Liying5, Enmai4, Yanan11, Dixiuzao, Taizhong23, Zhoumai27, Fengdecun5, Zhengmai366, Shannong14, Zhengmai101, and Niuzhijia) were identified, which had both high concentrations and high contents of mineral nutrients in the grain, and their high mineral concentrations are not related to small grain weight. Therefore, these outstanding wheat genotypes can be considered as promising donors for developing mineral-dense wheat cultivars.

### Antagonistic Relationship Between Mineral Nutrients

In this study and our previous study (Hao et al., 2021), the different trends in grain concentration of Cu, Mn, Zn, and Fe over the breeding period 1933–2017 were observed, which was mainly attributed to breeding effort in China through the breeding period (Hao et al., 2021). In addition, Zn and the other three mineral nutrients (Cu, Mn, and Fe) in plants generally displayed anantagonistic relationship (Chattha et al., 2017; Rehman et al., 2018), which might also be partly responsible for these different trends. It has been reported that enhanced Zn uptake in plants could reduce the absorption of Cu, Mn, and Fe in plants and thus lower accumulation in grains (Chattha et al., 2017; Rehman et al., 2018). In addition, a mutual antagonistic effect between Zn and P in plants has also been reported (Fageria, 2001), which might have contributed to different trends in grain Zn and P. It is found that high levels of P can increase biomass and thereby dilute Zn within the plant; on the other hand, Zn plays a key role in the regulation of P uptake, and high Zn could decrease the uptake and accumulation of P in the plant (Huang et al., 2000). Therefore, to alleviate Zn malnutrition in humans through either agronomic or genetic biofortification will require appropriate P fertilizer management (Chen et al., 2017). So far, the physiological mechanisms behind antagonistic relationship among mineral nutrients (e.g., Zn, Fe, Cu, and Mn) in wheat are still unclear; future studies are needed to evaluate the impact of antagonistic effect among these mineral nutrients on their uptake, translocation, and accumulation in wheat.

## Conclusion

Based on the results from a 2-year field study, a wide range of genetic variation in grain concentrations of mineral nutrients (Cu, Mn, N, P, and K) was identified among 292 tested wheat genotypes. Grain concentrations of Cu and Mn did not differ between Chinese landraces and cultivars, and cultivars possess a considerable genetic variation in grain Mn concentration, and the variation in grain Cu was slightly wide for landraces. The data showed that increased grain yield had a strong and negative effect on grain concentration of macronutrients (N, P, and K), but a relative weak and negative effect on micronutrients (Cu and Mn). Moreover, a yield level might be a main factor determining grain Cu concentration, and might only has a minor influence on grain Mn concentration. The interesting finding is, when considering the impact of variation in yield on mineral concentrations, wheat breeding in China over the past 80 years did not alter grain Mn and K concentrations, but it significantly decreased Cu, N, and P concentrations in the grain. Finally, in the current study, a total of 14 outstanding wheat accessions were identified, which had high-grain mineral nutrients concentrations/contents and can be considered as promising donors for developing mineral-dense wheat cultivars.

## Data Availability Statement

The raw data supporting the conclusions of this article will be made available by the authors, without undue reservation.

## Author Contributions

BH and LJ conceived the research. BH, XW, CY, LS, and SM designed and performed the experiments. BH and JM analyzed the data and drafted the manuscript. LJ, CL, and ZG provided substantial comments toward improving the content of the manuscript. LJ and ZW supervised the research. All authors read and approved the final copy of the manuscript.

## Conflict of Interest

The authors declare that the research was conducted in the absence of any commercial or financial relationships that could be construed as a potential conflict of interest.

## Publisher’s Note

All claims expressed in this article are solely those of the authors and do not necessarily represent those of their affiliated organizations, or those of the publisher, the editors and the reviewers. Any product that may be evaluated in this article, or claim that may be made by its manufacturer, is not guaranteed or endorsed by the publisher.
