# Supplementary material for: Did Wheat Breeding Simultaneously Alter Grain Concentrations of Macro- and Micro-Nutrient Over the Past 80 Years of Cultivar Releasing in China?
Source: Front Plant Sci. 2022 Mar 30;13:872781. doi: 10.3389/fpls.2022.872781 (PMC9009353; doi:10.3389/fpls.2022.872781)
Supplement: Supplementary file 1 [file Data_Sheet_1.PDF]

Supplementary Material

Supplementary table 1. Wheat landraces and cultivars used in this study

Code	Wheat line name	Year of release	Status	Code	Wheat line name	Year of release	Status
1	Jingyang60	1933	Cultivar	147	Shannong32	2016	Cultivar
2	Zhongnong28	1938	Cultivar	148	Shinong952	2016	Cultivar
3	Chixiaomai	1940	Cultivar	149	Bainong419	2017	Cultivar
4	Taizhong23	1941	Cultivar	150	Saide5	2017	Cultivar
5	Bima1	1942	Cultivar	151	Saide7	2017	Cultivar
6	Bima4	1942	Cultivar	152	Luohanmai26	2017	Cultivar
7	Nanda2419	1942	Cultivar	153	Xinkemai169	2017	Cultivar
8	Liying5	1946	Cultivar	154	Zhoumai33	2017	Cultivar
9	Nonglin10	1946	Cultivar	155	Hongpixiaomai		Landrace
10	Xinong 6028	1947	Cultivar	156	Dabaipi		Landrace
11	Triumph	1948	Cultivar	157	Xiaohongpi		Landrace
12	Atlas66	1950	Cultivar	158	Honglidangnianlao		Landrace
13	Early Premium	1950	Cultivar	159	Chunxiaomai		Landrace
14	Qianjiaomai	1950	Cultivar	160	Huoliaomai		Landrace
15	Shijiazhuang407	1950	Cultivar	161	Shanxibaimai		Landrace
16	Tanori	1950	Cultivar	162	Niuzhijia		Landrace
17	Aodesa3	1951	Cultivar	163	Mahuaban		Landrace
18	Gansu96	1952	Cultivar	164	Jiahongmai		Landrace
19	Gaojiasuo	1955	Cultivar	165	Hongjinmai		Landrace
20	Neixiang5	1955	Cultivar	166	Daimanghongmai		Landrace
21	Funo	1956	Cultivar	167	Honglaomai		Landrace
22	Nongda311	1956	Cultivar	168	Youmangbaifu		Landrace
23	Nongda183	1957	Cultivar	169	Hongpidongmai		Landrace
24	Dongnong101	1958	Cultivar	170	Panshiwumang		Landrace
25	Huadong6	1958	Cultivar	171	Youmangbaifu		Landrace
26	Shijiazhuang54	1958	Cultivar	172	Baiqiumai		Landrace
27	Shuwan8	1958	Cultivar	173	Xianmai		Landrace
28	Orofen	1959	Cultivar	174	Jiangxizao		Landrace
29	Jinan2	1960	Cultivar	175	Honghuazao		Landrace
30	Shuiyuan86	1960	Cultivar	176	Jiangdongmen		Landrace
31	Beijing8	1962	Cultivar	177	Dahuangpi		Landrace
32	Dingxi24	1963	Cultivar	178	Chongyanghongmai		Landrace
33	Fuzhuang30	1963	Cultivar	179	Zaowutian		Landrace
34	Fengchan3	1964	Cultivar	180	Liuzhutou		Landrace
35	Ganmai8	1964	Cultivar	181	Chanbuzhi		Landrace
36	Xiannong39	1964	Cultivar	182	Zhumaoyuanzitou		Landrace
37	Youbaomai	1964	Cultivar	183	Shuilizhan		Landrace
38	Dixiuzao	1965	Cultivar	184	Huangshuibai		Landrace

39	Zhengzhou6	1965	Cultivar	185	Baipu	Landrace
40	Emai6	1966	Cultivar	186	Zaoxiaomai	Landrace
41	Fengmai11	1966	Cultivar	187	Lanxizaoxiaomai	Landrace
42	Kangxiu10	1966	Cultivar	188	Wangshuibai	Landrace
43	Dongfanghong3	1967	Cultivar	189	Wuyuanmai	Landrace
44	Xinshuguang1	1967	Cultivar	190	Chejianzi	Landrace
45	Nongda139	1968	Cultivar	191	Heshangmai	Landrace
46	Fan6	1969	Cultivar	192	Rumai	Landrace
47	Hongmangmai	1969	Cultivar	193	Mangxiaomai	Landrace
48	Rikaze8	1969	Cultivar	194	Sankecun	Landrace
49	Tuokexun1	1969	Cultivar	195	Paozimai	Landrace
50	Yanan11	1969	Cultivar	196	Baiyoumai	Landrace
51	Aifeng3	1970	Cultivar	197	Yangmai	Landrace
52	Gaoyuan506	1970	Cultivar	198	Daqingmang	Landrace
53	Jichun1016	1970	Cultivar	199	Guangtou	Landrace
54	Kashi1	1970	Cultivar	200	Chaoanxiaomai	Landrace
55	Lvhan328	1970	Cultivar	201	Chike	Landrace
56	Huining10	1971	Cultivar	202	Songruimai	Landrace
57	Jinmai4	1971	Cultivar	203	Shengen	Landrace
58	Taishan1	1971	Cultivar	204	Shanglinxiaomai	Landrace
59	Zhemai1	1971	Cultivar	205	Baimangmai	Landrace
60	Jinmai2148	1972	Cultivar	206	Huangguaxian	Landrace
61	Lovrin10	1972	Cultivar	207	Banjiemang	Landrace
62	Rikaze54	1972	Cultivar	208	Laolaixia	Landrace
63	Xinshuguang6	1972	Cultivar	209	Louguding	Landrace
64	Zhengyin4	1972	Cultivar	210	Honggoudou	Landrace
65	Zangdong4	1973	Cultivar	211	Baihuomai	Landrace
66	Sumai3	1974	Cultivar	212	Sanyuehuang	Landrace
67	Zhengzhou741	1974	Cultivar	213	Hongqiangchang	Landrace
68	Bainong3217	1975	Cultivar	214	Youzimai	Landrace
69	Jimai2	1976	Cultivar	215	Pingyuan50	Landrace
70	Yannong15	1977	Cultivar	216	Baibiansui	Landrace
71	Qingchun28	1978	Cultivar	217	Baituzitou	Landrace
72	Enmai4	1980	Cultivar	218	Youmangsaogudan	Landrace
73	Xiaoyan6	1980	Cultivar	219	Qiangchangmai	Landrace
74	Xingyi4	1980	Cultivar	220	Huomai	Landrace
75	Yuandong822	1980	Cultivar	221	Meiqianwu	Landrace
76	Huzhuhong	1981	Cultivar	222	Jianmai	Landrace
77	Ningchun4	1981	Cultivar	223	Sanyuehuang	Landrace
78	Kefeng3	1982	Cultivar	224	Xiaofoshou	Landrace
79	Shannong7859	1982	Cultivar	225	Hongheshangtou	Landrace
80	Aimengniu	1983	Cultivar	226	Dakoumai	Landrace
81	Fengkang2	1983	Cultivar	227	Tumangmai	Landrace

82	Fengkang8	1983	Cultivar	228	Baitiaoyu	Landrace
83	Guinong10	1984	Cultivar	229	Baimangmai	Landrace
84	Xinkehan9	1984	Cultivar	230	Dayuhua	Landrace
85	Bimai26	1985	Cultivar	231	Fumai	Landrace
86	Kelao4	1985	Cultivar	232	Laoqimai	Landrace
87	Yunmai34	1985	Cultivar	233	Chushanbao	Landrace
88	Changzhi6406	1987	Cultivar	234	Zijiehong	Landrace
89	Kashibaipi	1990	Cultivar	235	Dalibanmang	Landrace
90	Pingyang27	1990	Cultivar	236	Liuyuehuang	Landrace
91	Jing411	1991	Cultivar	237	gejiaxiang	Landrace
92	Yangmai158	1993	Cultivar	238	Geerhongmai	Landrace
93	Laizhou953	1994	Cultivar	239	Bailanghuimai	Landrace
94	Wenmai6	1998	Cultivar	240	Bendihuanghuamai	Landrace
95	Jinan17	1999	Cultivar	241	Zhahong	Landrace
96	Shite14	2000	Cultivar	242	Motuoxiaomai	Landrace
97	Yanzhan1	2000	Cultivar	243	Bianbachunmai6	Landrace
98	Yumai49	2000	Cultivar	244	Baimangxiaomai	Landrace
99	Gan6172	2001	Cultivar	245	Wujiangzhuo	Landrace
100	Jimai19	2001	Cultivar	246	Muzongzhuoga	Landrace
101	Yannong19	2001	Cultivar	247	Kangdingxiaomai	Landrace
102	Zhengmai9023	2001	Cultivar	248	Shanmai	Landrace
103	Pingan11	2002	Cultivar	249	Yizhimai	Landrace
104	Anhui3	2003	Cultivar	250	Dabaimai	Landrace
105	Jimai20	2003	Cultivar	251	Galaohan	Landrace
106	Ankang58	2005	Cultivar	252	Huoliyan	Landrace
107	Xinong979	2005	Cultivar	253	Shanmai	Landrace
108	Zhengmai366	2005	Cultivar	254	Hongtuzi	Landrace
109	Gan7086	2006	Cultivar	255	Baidatou	Landrace
110	Shannong14	2006	Cultivar	256	Jinhuangmai	Landrace
111	Nongda211	2007	Cultivar	257	Baiqitou	Landrace
112	Shimai8	2007	Cultivar	258	Baimazha	Landrace
113	Shiyou17	2007	Cultivar	259	Laotutou	Landrace
114	Zhenmai168	2007	Cultivar	260	Tongjiabaxiaomai	Landrace
115	Liangxing66	2008	Cultivar	261	Honghuamai	Landrace
116	Zhoumai23	2008	Cultivar	262	Baimaizi	Landrace
117	Liangxing77	2010	Cultivar	263	Chengduguangtou	Landrace
118	Shannong20	2010	Cultivar	264	Jiangmai	Landrace
119	Mingxian169	2011	Cultivar	265	Baihuamai	Landrace
120	Pingan8	2011	Cultivar	266	Huangxiangguo	Landrace
121	Shannong23	2011	Cultivar	267	Hanzhongbai	Landrace
122	Shimai19	2011	Cultivar	268	Xiaosanyuehuang	Landrace
123	Shimai22	2011	Cultivar	269	Suotianhongmai	Landrace
124	Zhengmai7698	2011	Cultivar	270	Hongxumai	Landrace

125	Zhoumai27	2011	Cultivar	271	Zipi	Landrace
126	Zhongmai895	2012	Cultivar	272	Baimangmai	Landrace
127	Zhengmai101	2013	Cultivar	273	Hongmangzi	Landrace
128	Bainong210	2013	Cultivar	274	Yuqiumai	Landrace
129	Bainong207	2013	Cultivar	275	Yangmai	Landrace
130	Nongda399	2012	Cultivar	276	Yangmai	Landrace
131	Zhoumai28	2013	Cultivar	277	Zhushimai	Landrace
132	Annong0711	2014	Cultivar	278	Biantouguangkemai	Landrace
133	Qimai2	2014	Cultivar	279	Changmangshibiantou	Landrace
134	Shinong086	2014	Cultivar	280	Zhugoumai	Landrace
135	Tianmin197	2014	Cultivar	281	Dianxihongkeyangmai	Landrace
136	Tianmin198	2014	Cultivar	282	Baidongmai	Landrace
137	Xinmai26	2014	Cultivar	283	Hongchunmai	Landrace
138	Yumai158	2014	Cultivar	284	Chunmai	Landrace
139	Yangfumai101	2014	Cultivar	285	Hongdongmai	Landrace
140	Zhengmai119	2014	Cultivar	286	Hongdongmai	Landrace
141	Fengdecun5	2015	Cultivar	287	Wumangchunmai	Landrace
142	Denghaihongdi95	2016	Cultivar	288	Hongchunmai	Landrace
143	Jimai23	2016	Cultivar	289	Hongjinbaoying	Landrace
144	Jimai229	2016	Cultivar	290	Hongdongmai	Landrace
145	Jimai262	2016	Cultivar	291	Wumangchunmai	Landrace
146	Shannong29	2016	Cultivar	292	Chinese Spring	Landrace

Notes: Information of wheat lines were mainly based on the following references, and integrated with data from Chinese Crop Germplasm Information System (CGRIS), Chinese Academy of Agricultural Sciences (CAAS) (http://www.cgris.net).

Jin, S.B., and Liu, A.D., Wheat in China. Beijing: Chinese Agriculture Press, 1964 (in Chinese)
Jin, S.B., Wheat in China (1962-1982). Beijing: Chinese Agriculture Press, 1986 (in Chinese)
Jin, S.B., Wheat in China (1983-1993). Beijing: Chinese Agriculture Press, 1997 (in Chinese)
Zhuang, Q.S., Improvement and pedigree of China wheat varieties. Beijing: China Agriculture Press, 2003 (in Chinese)

Supplementary Table 2. The top 20 genotypes for grain mineral nutrients concentration (Zn, Fe, Cu, Mn, N, P and K) in the 2018 and 2019 season and average of the two seasons

Zn (mg kg^{-1})

Rank	2018 season			2019 season			Average the two seasons		
	Line code	Wheat line name	Grain Zn concentration	Line code	Wheat line name	Grain Zn concentration	Line code	Wheat line name	Grain Zn concentration
1	276	Yangmai	67.25	43	Dongfanghong3	79.26	160	Huoliaomai	63.25
2	287	Wumangchunmai	61.79	4	Taizhong23	74.12	90	Pingyang27	62.28
3	176	Jiangdongmen	60.95	160	Huoliaomai	71.61	276	Yangmai	59.88
4	25	Huadong6	55.07	90	Pingyang27	70.15	4	Taizhong23	59.40
5	160	Huoliaomai	54.89	128	Bainong210	62.07	176	Jiangdongmen	58.52
6	190	Chejianzi	54.62	253	Shanmai	59.59	253	Shanmai	56.16
7	250	Dabaimai	54.52	150	Saide5	57.94	43	Dongfanghong3	55.89
8	90	Pingyang27	54.42	59	Zhemai1	57.38	128	Bainong210	54.79
9	194	Sankecun	54.41	139	Yangfumai10	56.77	139	Yangfumai10	54.38
10	66	Sumai3	54.16	170	Panshiwumang	56.42	185	Baipu	53.41
11	198	Daqingmang	53.17	208	Laolaixia	56.40	159	Chunxiaomai	53.22
12	253	Shanmai	52.73	176	Jiangdongmen	56.09	287	Wumangchunmai	53.08
13	251	Galaohan	52.67	175	Honghuazao	55.91	175	Honghuazao	53.00
14	139	Yangfumai10	51.98	143	Jimai23	55.36	143	Jimai23	52.86
15	257	Baiqitou	51.86	185	Baipu	55.22	150	Saide5	52.67
16	185	Baipu	51.61	159	Chunxiaomai	55.06	190	Chejianzi	52.31
17	159	Chunxiaomai	51.38	98	Yumai49	55.05	170	Panshiwumang	52.30
18	143	Jimai23	50.35	264	Jiangmai	54.83	162	Niuzhijia	51.92
19	175	Honghuazao	50.08	171	Youmangbaifu	54.54	25	Huadong6	51.37
20	162	Niuzhijia	50.08	211	Baihuomai	54.52	140	Zhengmai119	51.24

Fe (mg kg^{-1})

Rank	2018 season			2019 season			Average the two seasons		
	Line code	Wheat line name	Grain Fe concentration	Line code	Wheat line name	Grain Fe concentration	Line code	Wheat line name	Grain Fe concentration
1	207	Banjiemang	59.80	160	Huoliaomai	85.12	160	Huoliaomai	66.98
2	155	Hongpixiaomai	59.71	88	Changzhi6406	83.18	207	Banjiemang	63.62
3	159	Chunxiaomai	58.55	50	Yanan11	78.81	88	Changzhi6406	62.43
4	251	Galaohan	58.20	207	Banjiemang	67.43	159	Chunxiaomai	58.76
5	239	Bailanghuimai	57.11	31	Beijing8	62.85	50	Yanan11	58.69
6	198	Daqingmang	57.10	4	Taizhong23	60.96	219	Qiangchangmai	58.10
7	219	Qiangchangmai	56.25	81	Fengkang2	60.04	155	Hongpixiaomai	57.96
8	62	Rikaze54	55.78	219	Qiangchangmai	59.95	239	Bailanghuimai	56.39
9	253	Shanmai	54.99	159	Chunxiaomai	58.97	62	Rikaze54	54.54
10	250	Dabaimai	54.41	59	Zhemai1	57.97	217	Baotuzitou	54.49
11	217	Baituzitou	53.25	43	Dongfanghong3	57.41	198	Daqingmang	54.12
12	257	Baiqitou	53.04	155	Hongpixiaomai	56.21	81	fengkang2	54.06
13	276	Yangmai	52.03	217	Baituzitou	55.73	231	Fumai	53.25
14	231	Fumai	51.65	239	Bailanghuimai	55.68	162	Niuzhijia	52.62
15	176	Jiangdongmen	51.49	231	Fumai	54.84	4	Taizhong23	52.25
16	243	Bianbachunmai6	51.38	162	Niuzhijia	54.10	31	Beijing8	51.67
17	206	Huangguaxian	51.18	62	Rikaze54	53.31	170	Panshiwumang	50.91
18	162	Niuzhijia	51.13	185	Baipu	52.57	185	Baipu	50.57
19	90	Pingyang27	51.06	98	Yumai49	52.55	243	Bianbachunmai6	50.48
20	170	Panshiwumang	50.93	119	Mingxian169	52.21	220	Huomai	50.17

Cu (mg kg^{-1})

Rank	2018 season			2019 season			Average the two seasons		
	Line code	Wheat line name	Grain Cu concentration	Line code	Wheat line name	Grain Cu concentration	Line code	Wheat line name	Grain Cu concentration

1	286	Hongdongmai	9.90	6	Bima4	9.01	286	Hongdongmai	9.25
2	38	Dixiuzao	9.39	4	Taizhong23	8.78	38	Dixiuzao	8.47
3	253	Shanmai	9.02	286	Hongdongmai	8.60	59	Zhemai1	8.47
4	231	Fumai	8.86	50	Yanan11	8.31	50	Yanan11	8.33
5	59	Zhemai1	8.69	59	Zhemai1	8.24	231	Fumai	8.28
6	213	Hongqiangchang	8.49	212	Sanyuehuang	7.89	4	Taizhong23	8.18
7	198	Daqingmang	8.41	231	Fumai	7.69	212	Sanyuehuang	7.96
8	8	Liying5	8.34	39	Zhengzhou6	7.55	213	Hongqiangchang	7.91
9	50	Yanan11	8.34	23	Nongda183	7.43	72	Enmai4	7.82
10	72	Enmai4	8.28	160	Huoliaomai	7.36	207	Banjiemang	7.74
11	54	Kashi1	8.23	72	Enmai4	7.35	8	Liying5	7.70
12	207	Banjiemang	8.14	207	Banjiemang	7.34	253	Shanmai	7.69
13	40	Emai6	8.12	213	Hongqiangchang	7.33	23	Nongda183	7.45
14	104	Anhui3	8.12	8	Liying5	7.06	163	Mahuaban	7.32
15	212	Sanyuehuang	8.04	205	Baimangmai	7.01	47	Hongmangmai	7.29
16	210	Honggoudou	7.85	163	Mahuaban	6.87	205	Baimangmai	7.28
17	163	Mahuaban	7.77	47	Hongmangmai	6.83	104	Anhui3	7.26
18	55	Lvhan328	7.77	43	Dongfanghong3	6.82	210	Honggoudou	7.18
19	47	Hongmangmai	7.76	75	Yuandong822	6.73	75	Yuandong822	7.18
20	169	Hongpidongmai	7.66	239	Bailanghuimai	6.72	169	Hongpidongmai	7.16

**Mn (mg kg^{-1})**									
**Rank**	**2018 season**			**2019 season**			**Average the two seasons**		
	**Line code**	**Wheat line name**	**Grain Mn concentration**	**Line code**	**Wheat line name**	**Grain Mn concentration**	**Line code**	**Wheat line name**	**Grain Mn concentration**
1	38	Dixiuzao	51.77	4	Taizhong23	57.49	4	Taizhong23	53.65
2	4	Taizhong23	49.80	38	Dixiuzao	51.98	38	Dixiuzao	51.88
3	40	Emai6	48.28	96	Shite14	47.26	141	Fengdecun5	47.23
4	141	Fengdecun5	47.88	141	Fengdecun5	46.57	241	Zhahong	45.40

5	108	Zhengmai366	45.63	278	Biantouguangkemai	46.54	108	Zhengmai366	45.15
6	125	Zhoumai27	45.06	241	Zhahong	46.01	125	Zhoumai27	44.96
7	241	Zhahong	44.78	125	Zhoumai27	44.87	278	Biantouguangkemai	44.40
8	16	Tanori	44.32	108	Zhengmai366	44.68	96	Shite14	44.38
9	204	Shanglinxiaomai	44.05	204	Shanglinxiaomai	44.44	204	Shanglinxiaomai	44.24
10	90	Pingyang27	43.81	16	Tanori	44.07	16	Tanori	44.20
11	127	Zhengmai101	43.70	189	Wuyuanmai	43.41	127	Zhengmai101	43.46
12	162	Niuzhijia	43.67	127	Zhengmai101	43.21	189	Wuyuanmai	43.37
13	160	Huoliaomai	43.34	162	Niuzhijia	43.07	162	Niuzhijia	43.37
14	279	Changmangshibiantou	43.34	200	Chaoanxiaomai	43.00	200	Chaoanxiaomai	43.06
15	189	Wuyuanmai	43.33	279	Changmangshibiantou	42.71	279	Changmangshibiantou	43.02
16	5	Bima1	43.22	277	Zhushimai	42.29	40	Emai6	42.39
17	200	Chaoanxiaomai	43.11	1	Jingyang60	41.43	110	Shannong14	41.76
18	110	Shannong14	42.53	110	Shannong14	40.98	1	Jingyan60	41.73
19	278	Biantouguangkemai	42.27	114	Zhenmai168	40.87	3	Chixiaomai	41.52
20	4	Taizhong23	42.24	3	Chixiaomai	40.81	114	Zhenmai168	40.89

N, P and K (mg g^{-1})

Rank	2018 season			2018 season			2018 season		
	**Line code**	**Wheat line name**	**Grain N concentration**	**Line code**	**Wheat line name**	**Grain P concentration**	**Line code**	**Wheat line name**	**Grain K concentration**
1	253	Shanmai	43.10	253	Shanmai	7.52	89	Kashibaipi	8.72
2	231	Fumai	39.79	231	Fumai	6.12	253	Shanmai	7.87
3	89	Kashibaipi	37.94	239	Bailanghuimai	6.00	278	Biantouguangkemai	7.48
4	278	Biantouguangkemai	36.70	89	Kashibaipi	5.98	207	Banjiemang	7.08
5	243	Bianbachunmai6	36.58	236	Liuyuehuang	5.94	239	Bailanghuimai	6.84
6	271	Zipi	36.36	252	Huoliyan	5.71	62	Rikaze54	6.81
7	213	Hongqiangchang	36.35	251	Galaohan	5.71	280	Zhugoumai	6.44
8	292	China Spring	35.69	276	Yangmai	5.65	245	Wujiangzhuo	6.41

9	204	Shanglinxiaomai	35.39	286	Hongdongmai	5.61	288	Hognchunmai	6.26
10	270	Hongxumai	35.02	207	Banjiemang	5.46	47	Hongmangmai	6.20
11	47	Hongmangmai	34.98	278	Biantouguangkemai	5.45	283	Hongchunmai	6.08
12	207	Banjiemang	34.63	76	Huzhuhong	5.41	292	China Spring	6.07
13	279	Changmangshibiantou	34.62	237	Gejiaxiang	5.41	67	Zhengzhou741	6.07
14	269	Suotiaohongmai	34.48	62	Rikaze54	5.37	231	Fumai	6.07
15	76	Huzhuhong	34.22	284	Chunmai	5.36	77	Ningchun4	6.06
16	284	Chunmai	33.32	279	Changmangshibiantou	5.29	286	Hongdongmai	6.04
17	237	Gejiaxiang	33.06	270	Hongxumai	5.28	279	Changmangshibianmai	5.99
18	68	Bainong3217	32.95	67	Zhengzhou741	5.27	265	Baihuamai	5.96
19	217	Baituzitou	32.73	265	Baihuamai	5.22	65	Zangdong4	5.94
20	198	Daqingmang	32.42	283	Hongchunmai	5.16	57	Jinmai4	5.89
